# Dr. George W. Lewis

**Published:** 1900-08

**Authors:** 


Dr. George W. Lewis, of Buffalo, died at his home, 311 Delaware Avenue, July 24, 1900, aged 38 years. His death was sudden, having occurred at about 1 o'clock a. m. He went to bed a little before midnight in apparent health. He was one of the prominent younger homeopathic physicians and will be sadly missed among his large circle of friends. He was a nephew of Judge Loran L. Lewis, and his father, the late Dr. George W. Lewis, was one of a group of homeopathic physicians that were prominent for many years in this city.

Dr. Lewis attended the public schools and high schools here, and graduated at Cornell University in 1884. He graduated in medicine at a New York Homeopathic College, spent some time abroad in study and has been engaged in practice nearly fifteen years. He leaves a widow and two children.

